# Associations of White Matter and Basal Ganglia Microstructure to Cognitive Fatigue Rate in Multiple Sclerosis

**DOI:** 10.3389/fneur.2022.911012

**Published:** 2022-07-04

**Authors:** Cristina A. F. Román, Glenn R. Wylie, John DeLuca, Bing Yao

**Affiliations:** ^1^Rocco Ortenzio Neuroimaging Center, Kessler Foundation, West Orange, NJ, United States; ^2^Department of Physical Medicine and Rehabilitation, Rutgers New Jersey Medical School, New Jersey, NJ, United States; ^3^Department of Veterans Affairs, The War Related Illness and Injury Center, New Jersey Healthcare System, East Orange, NJ, United States; ^4^Department of Neurology, Rutgers New Jersey Medical School, Newark, NJ, United States

**Keywords:** multiple sclerosis (MS), cognitive fatigue, diffusional kurtosis imaging (DKI), white matter, basal ganglia, microstructure

## Abstract

Fatigue, including cognitive fatigue, is one of the most debilitating symptoms reported by persons with multiple sclerosis (pwMS). Cognitive fatigue has been associated with disruptions in striato-thalamo-cortical and frontal networks, but what remains unknown is how the *rate* at which pwMS become fatigued over time relates to microstructural properties within the brain. The current study aims to fill this gap in knowledge by investigating how cognitive fatigue rate relates to white matter and basal ganglia microstructure in a sample of 62 persons with relapsing-remitting MS. Participants rated their level of cognitive fatigue at baseline and after each block (x7) of a within-scanner cognitive fatigue inducing task. The slope of the regression line of all eight fatigue ratings was designated as “cognitive fatigue rate.” Diffusional kurtosis imaging maps were processed using tract-based spatial statistics and regional analyses (i.e., basal ganglia) and associated with cognitive fatigue rate. Results showed cognitive fatigue rate to be related to several white matter tracts, with many having been associated with basal ganglia connectivity or the previously proposed “fatigue network.” In addition, cognitive fatigue rate was associated with the microstructure within the putamen, though this did not survive multiple comparisons correction. Our approach of using cognitive fatigue rate, rather than trait fatigue, brings us closer to understanding how brain pathology may be impacting the experience of fatigue in the moment, which is crucial for developing interventions. These results hold promise for continuing to unpack the complex construct that is cognitive fatigue.

## Introduction

Fatigue, including cognitive fatigue (i.e., lack of mental energy), is one of the most widely reported symptoms in multiple sclerosis (MS), impacting more than 70–90% of individuals with the disease (1, 2). The presence and severity of fatigue negatively impacts employment, quality of life, psychological status, and ability to complete basic and complex activities of daily living (3–5). What we know about cognitive fatigue to date, however, has largely stemmed from subjective self-report inventories, which rely on retrospective ratings and carry several limitations. There is evidence that measuring fatigue in the moment (i.e., state fatigue) may provide a more accurate measure, as it is less contaminated by outside factors such as bias, memory, and mood state (6).

Adding to the complexity and nuance of measuring cognitive fatigue is the absence of a metric that tracks change over time, or how quickly a person with MS becomes fatigued during a cognitively demanding task (i.e., *rate* of cognitive fatigue). Studies examining cognitive fatigue rate in MS are limited, but we can deduce the importance of considering rate through the *temporal fatigue hypothesis*. The *temporal fatigue hypothesis* posits that there is a positive relationship between mental effort and subjective cognitive fatigue, regardless of cognitive load, such that as length of time engaged in a mentally demanding task increases, so does level of reported subjective cognitive fatigue (7–9). Several studies have found increases in subjective cognitive fatigue in relation to time spent engaging in a cognitively demanding task, with little or no association found between subjective cognitive fatigue and performance (10–12). No studies have directly investigated the *rate* at which persons with MS fatigue while engaging in a fatigue inducing task. The current study aims to fill this gap by examining the rate of cognitive fatigue over time during a fatigue inducing task.

Investigations of cognitive fatigue and white matter microstructure using diffusion weighted imaging (DWI) have produced potential white matter correlates of cognitive fatigue that appear to be consistent with the striato-thalamo-cortical network proposed by multiple investigators [see (13–16)]. In separate studies examining persons with MS, fibers connecting the posterior hypothalamus and mesencephalon, external capsule, internal capsule, frontal and occipital juxtacortical fibers, uncinate fasciculus, forceps minor, superior longitudinal fasciculus, and cingulum have all been associated with trait fatigue (17–19). Reduced striato-thalamo-cortical and frontal network integrity have also been associated with cognitive fatigue in veterans with a history of mild to moderate traumatic brain injury and older adults (20, 21).

In addition to the contribution of white matter damage to cognitive fatigue in neurological (i.e., MS) and non-neurological populations, brain structures, particularly the basal ganglia, have also shown associations. Chaudhuri and Behan (13) were among the first to propose that the basal ganglia are implicated in cognitive fatigue due to interruptions of basal ganglia circuitry (i.e., striato-thalamo-cortical loop). Subsequent work using neuroimaging has supported this hypothesis by linking the structure and function of the basal ganglia to both cognitive and general fatigue in MS [e.g., (22, 23)]. Additional studies in MS (14, 24–28) and non-MS populations (29–32) further support the basal ganglia as a primary pathophysiological contributor to fatigue.

Previous studies have linked cognitive fatigue and changes to white matter and/or basal ganglia structure, but limitations exist. First, most studies have relied on trait fatigue as the primary independent/dependent variable, which has limited accuracy due to retrospective self-report biases. Second, no previous studies have taken *rate* of fatigue into account, thereby missing a crucial aspect of cognitive fatigue. Lastly, while basal ganglia activation/connectivity and overall volume have been examined, no previous studies have utilized advanced DWI to examine the microstructure of the basal ganglia. The current study aims to fill these gaps in the current literature by examining the relationship between cognitive fatigue rate (i.e., how quickly or slowly an individual becomes cognitively fatigued during a fatigue inducing task) and white matter and basal ganglia microstructure using advanced DWI.

## Methods

### Participants

The current study represents secondary analyses on a previously collected prospective dataset. Seventy-three participants with clinically definite relapsing-remitting MS (RRMS) according to McDonald criteria (33) were recruited for the study. Eleven participants were not included due to incomplete study sessions or substantially missing behavioral data (i.e., did not come in for scheduled session, etc.), leaving 62 participants enrolled in the study. Of the 62 participants, six were missing or had unusable neuroimaging data and were thus excluded from the neuroimaging portion of the study. There were no demographic or neuropsychological differences between the participants included and excluded from neuroimaging analyses, aside from years of education completed. The participants included in neuroimaging analyses had more years of education (M = 16.13, SD = 1.71) than those who were excluded (M = 14.50, SD = 2.51, *p* = 0.038). Table 1 provides demographic characteristics for the study sample.

**Table 1:** Descriptive characteristics of demographic and behavioral data.

	**Sample (*N* = 62) *n* (%)**
Age	52.2 ± 8.5, 54 (30–66)
Female	50 (80.6%)
Race/ethnicity	
Latinx/hispanic	7 (11.3%)
Afro-Latinx	1 (1.6%)
Non-Latinx Black	4 (6.5%)
White	39 (62.9%)
Asian	1 (1.6%)
Other	9 (14.5%)
Not reported	1 (1.6%)
Years of education	16.0 ± 1.8, 16.0 (12–20)
Disease Duration^*^	19.1 ± 10.8, 17.5 (2–43)
Lesion Volume (mL)	6.4 ± 7.6, 3.1 (0–40.1)
MFIS Cognitive	18.8 ± 8.9, 17.0 (0–39)
MFIS Psychological	3.39 ± 2.16, 3.0 (0–8)
MFIS Physical	17.73 ± 8.0, 19.0 (0–35)
MFIS Total	37.9 ± 16.1, 38.0 (4–75)
CMDI Mood	8.08 ± 3.68, 6.0 (6–25)
CMDI Evaluative	14.02 ± 6.10, 13.0 (1–42)
CMDI Vegetative	25.53 ± 6.64, 24.50 (11–40)
CMDI Total	77.2 ± 23.8, 70.1 (45–181)
STAI State	31.8 ± 10.5, 28.0 (20–63)
STAI Tait	36.2 ± 11.6, 34.0 (21–61)
SDMT Raw	50.9 ± 12.8, 52.0 (17–74)

* *Missing data for four participants; MFIS, Modified Fatigue Impact Scale; CMDI, Chicago Multiscale Depression Inventory; STAI, State Trait Anxiety Inventory; SDMT, Symbol Digit Modalities Test*.

RRMS participants were recruited from local universities and MS clinics, flyers posted throughout the community and on MS-related websites, ads placed within local MS chapter newsletters, and from a database of over 500 MS participants who have participated in research at our institution in the past. Inclusion criteria for the MS group were as follows: between 30–65 years of age; RRMS subtype (verified by each participant's neurologist); free of exacerbations for at least 1 month prior to the screening; and able to ambulate without an assistive device. Exclusion criteria were as follows: history of head injury, stroke, seizures, or any other neurological history outside of MS; current treatment/use of steroids, benzodiazepines, antipsychotics, and/or neuroleptics (i.e., at the time of the phone screen or study session); unable or unwilling to consent; and contraindications for MRI. All prospective participants underwent a telephone screen to determine eligibility, and eligible participants were scheduled for an in-person study session which included consenting, completion of questionnaires, neurocognitive testing, and MRI. All participants were compensated for their time ($100 USD). All study procedures were conducted in English and were approved by the Kessler Foundation Institutional Review Board. Each participant received the same battery and administration was standardized such that the order of the battery was kept consistent across participants.

### Behavioral Measures

Each participant completed a set of questionnaires measuring depression, state and trait anxiety, and trait fatigue. These variables were examined to better understand the variance of cognitive fatigue rate.

#### Chicago Multiscale Depression Inventory

The Chicago Multiscale Depression inventory [CMDI; (34)] is a 50-item inventory consisting of four subscales: mood (14 items), evaluative (14 items), vegetative (14 items), and positive affect (eight items). These subscales can be used separately or in combination with one another. Participants rate themselves on a 5-point Likert scale (1- “Not at all” to 5-“Extremely”) the extent to which each word/phrase (e.g., sad, joyful, unworthy, gloomy) describes them “during the past week, including today.” The CMDI was designed specifically for use in medical populations, including MS. Raw scores for the mood, evaluative, and vegetative subscales and the total score were used in statistical analyses.

#### State-Trait Anxiety Inventory

The State-Trait Anxiety Inventory [STAI; (35)] is a 40-item measure divided into two, 20-item scales to assess current (“state”; e.g., “I am tense,” “I am worried”) and longstanding (“trait”; e.g., “I am content,” “I am a steady person”) anxiety. Participants rate themselves on a 4-point Likert scale (state: 1- “Not at all” to 4- “Very much so”; trait: 1- “Almost never” to 4- “Almost always”) based on how they feel in the moment (“state”) and how they generally feel in their lives (“trait”). Raw scores for state and trait anxiety were used in statistical analyses.

#### Modified Fatigue Impact Scale

The Modified Fatigue Impact Scale (MFIS) is a 21-item self-report questionnaire based on Fisk et al.'s (36) Fatigue Impact Scale. Items makeup three subscales that measure the effects of fatigue on cognitive (10 items; e.g., “I have been less alert”), physical (9 items; e.g., “I have had to pace myself in my physical activities”), and psychosocial (2 items; e.g., “I have been less motivated to participate in social activities”) functioning. Participants rate themselves on a 5-point Likert scale (0- “Never” to 4- “Almost always”) the extent to which fatigue has impacted them in the stated way during the previous 4 weeks. Raw scores for cognitive, physical, and psychosocial subscales were used in statistical analyses.

### Fatigue Induction Task

We used the same fatigue induction task that we have used in previous research (18, 37). On every trial, subjects were presented with a rotating, colored rectangle. The rectangle was rotating either quickly or slowly and was colored either red or blue. The stimulus on each trial therefore afforded two tasks: a color categorization task in which subjects pressed one button on an MR-compatible button box if the rectangle was colored red and another if it was colored blue; and a speed categorization task in which subjects pressed one button if the stimulus was rotating quickly and another if it was rotating slowly. The color and speed trials were optimized for deconvolution and were pseudo-randomly mixed such that on some trials subjects switched from one task to the other while on others they repeatedly performed each task. E-Prime software was used to present the stimuli and to record responses. Subjects worked through seven blocks of the task-switching paradigm to induce fatigue.

#### Visual Analog Scale-Fatigue

Participants' cognitive fatigue was assessed with a visual analog scale (VAS) at baseline and after each block of the fatigue induction task for a total of eight ratings. Participants were asked: “How tired are you right now?” and were asked to indicate their level of fatigue on a scale from 0 to 100, with 0 being minimally fatigued and 100 being maximally fatigued. To mask the purpose of the study, three additional VAS ratings were also administered (in randomized order) before and after each task block: happiness, sadness, and frustration. The slope of the regression line for each participant's eight VAS-F ratings was operationalized as “cognitive fatigue rate.”

#### Neuroimaging Acquisition

Neuroimaging data collection was completed on a 3-Tesla Siemens Skyra scanner. Data were collected using 20- and 32-channel head coils. Diffusion weighted imaging (DWI) data were collected A>>P using two separate sequences which were optimized to produce comparable data (sequence 1: *b* = 1,000, 2,000 s/mm,^2^ TR = 5,600 ms, TE = 97 ms, FOV = 220 mm, voxel size = 2.3 × 2.3 × 3.0 mm^3^, multi-band acceleration factor = none, TA= 6 min 50 s; sequence 2: *b* = 1,000, 2,000 s/mm,^2^ TR = 3,000 ms, TE = 95 ms, FOV = 220 mm, voxel size = 2.3 × 2.3 × 3.0 mm^3^, multi-band acceleration factor = 2, TA = 3 min 46 s). In addition, high-resolution magnetization prepared rapid gradient echo (MPRAGE) and T2 fluid attenuated inversion recovery (T2 FLAIR) images were acquired for each participant to quantify lesion volume (MPRAGE: TE = 3.43 ms; TR = 2,100 ms, FOV = 256 mm; flip angle = 9°; slice thickness = 1 mm, voxel = 1 × 1 × 1 mm^3^, matrix = 256 × 256, in-plane resolution = 1 mm^3^ isoptropic; T2 FLAIR: TE = 91 ms; TR = 9,000 ms, FOV = 256 mm; flip angle = 150°; slice thickness = 3 mm, voxel = 1 × 1 × 3 mm^3^, matrix = 256 × 216, in-plane resolution = 1 × 1 × 3 mm^3^).

### Lesion Quantification

White matter lesions were quantified using the Lesion Segmentation Toolbox v3.0.0 (LST) in Statistical Parametric Mapping 12 (SPM12), developed by Schmidt et al. (38). Prior to implementing the Lesion Segmentation Toolbox, each participant's T1-weighted and T2 FLAIR-weighted scans were visually inspected for artifacts and distortions. Six participants' data were poor quality and unusable. Missing data for these participants was imputed using Multiple Imputation by Chained Equations (MICE), which uses an iterative series of predictive models to ‘fill in' missing data (39).

The lesion growth algorithm (LGA) option within the LST was used to quantify lesions. In brief, T1-weighted images were segmented into three different tissue classification maps: gray matter (GM), white matter (WM), and cerebrospinal fluid (CSF). The T2 FLAIR-weighted images were bias-corrected and coregistered to the T1-weighted image. Next, the FLAIR intensity distribution for each tissue classification map was obtained and FLAIR-hyperintense outliers, representing sclerotic lesions, were added together to create a combined conservative lesion belief map. The conservative lesion belief map of each participant underwent an iterative process using a lesion growth model. During this process, each voxel within the neighborhood of a conservatively identified lesion was labeled as “lesion” or “other” depending on whether voxels share a common border or not; the lesion growth algorithm assumes that voxels that are completely surrounded by lesion voxels are more likely to represent lesions. The program then moves from conservative assumptions about the lesion map to more liberal assumptions by weighting the likelihood of a voxel belonging to gray or white matter vs. lesions. This process is enhanced by a hidden MRF segmentation model and a priori knowledge of the location of white matter (38). The final outputted lesion maps were used to quantify whole brain lesion volume in milliliters (mL) for each participant.

### Diffusion Weighted Imaging

All diffusion weighted imaging data was visually inspected for gross artifacts. As noted above, six participants were missing or had unusable imaging data and were thus excluded from the diffusion weighted imaging portion of the study. Diffusion data was preprocessed using PyDesigner's standard pipeline which integrates packages from FMRIB Software Library (FSL), MRtrix3, and Python (40–49). Preprocessing steps included denoising, Gibb's ringing correction, EPI distortion correction, eddy current correction, co-registration, brain mask computation (0.20 threshold), smoothing (FWHM = 1.25), and Rician bias correction. A Diffusional Kurtosis Imaging (DKI) model was applied to the data to produce DKI maps for mean kurtosis (MK), axial kurtosis (AK), and radial kurtosis (RK). A map for fractional anisotropy (FA) was also created and used as a reference when need. All DKI maps were checked for artifacts, intensity range problems, and general data quality. These maps were used to conduct tract-based spatial statistics [TBSS; (50)] analyses within FMRIB Software Library [FSL; (51)].

For TBSS, all participants' FA maps were put into a higher-resolution standard space using FSL's Non-linear Image Registration Tool [FNIRT; (64)]. First, a study-specific “target image” was created by aligning every FA image to every other one and then identifying the “most representative” image. The target image was then aligned into 1 × 1 × 1 mm MNI152 space using a combined non-linear transform and affine transform. Each participant's FA image was then aligned to this target. The mean of all FA images was calculated and thinned to create an FA skeleton, which encompasses the centers of all the white matter tracts common to the sample. The threshold for the FA skeleton was set to 0.2. This threshold value was chosen because it has been established as an appropriate threshold for segmenting white matter and gray matter (52). Prior to running the voxel wise cross-subject statistics, all aligned FA images were quality checked to ensure that there were no errors in registration, the FA skeleton was appropriately thresholded, and that each threshold within the FA skeleton could be matched to a white matter tract for each participant. Individual FA maps were then projected onto the mean FA skeleton. Once the reference FA skeleton was created, the “non-FA images” pipeline was used to apply TBSS to DKI maps (i.e., MK, AK, and RK).

### Basal Ganglia Microstructure

The Harvard-Oxford subcortical atlas (53–56) was used to create masks for basal ganglia structures, including the right/left caudate, pallidum, and putamen. The structures were extracted and binarized using FSL's “fslmaths” function separately for the right and left sides, resulting in six separate masks in standard space (i.e., right caudate, left caudate, right pallidum, left pallidum, right putamen, left putamen). To account for partial volume effects, each mask was binarized using FSL and eroded by one voxel (i.e., −1) using Analysis of Functional NeuroImage's [AFNI; (57)] “dilate” function. To create the transformation matrices needed to transform the ROIs into each participant's native diffusion (i.e., MK, RK, AK) space, each participant's FA map underwent linear, followed by non-linear transformations using FMRIB's Linear Image Registration Tool [FLIRT; (58, 59)] and FNIRT. Then, FSL's “invwarp” was used to create an inverse warped coefficient using the warp coefficient image generated by FNIRT. Finally, FSL's “applywarp” was run to put each participant's basal ganglia ROI mask into their native diffusion space. Quality checking occurred after each step within the pipeline to ensure all data were of good quality and without egregious artifacts/errors. Left and right caudate, pallidum, and putamen ROIs were combined to create single caudate, pallidum, and putamen masks for each participant. Each participant's binary caudate, pallidum, and putamen masks were then multiplied against each of their DKI maps (i.e., MK, AK, RK). Mean MK, AK, and RK values were then pulled from each of these basal ganglia structure x DKI maps using FSL's “fslstats” function. These mean MK, AK, and RK values were used in all analyses.

### Statistical Analyses

#### Demographics

For the analysis of demographic variables, SPSS Statistics (v28) was used to conduct basic descriptive and frequency analyses on age, sex, disease duration, lesion load, race/ethnicity, and education. Expanded Disability Status Scale (EDSS) and current disease modifying treatment status were unavailable. All covariates for subsequent analyses were chosen apriori based on previous studies and availability, as such covariates vary based on the dependent variable of interest to ensure we accounted for the most pertinent confounds.

#### Fatigue Induction Task and Cognitive Fatigue Rate

Behavioral data from the fatigue induction task (i.e., RT, accuracy) and cognitive fatigue rate were inspected for normality. Only RT and accuracy were found to be skewed, and they were transformed using the Box-Cox method to ensure that assumptions of normality were not violated (60). Linear regression analyses were conducted with cognitive fatigue rate as the independent variable and RT and accuracy as the dependent variable (in separate analyses). Sex, age, disease duration, and education were included in the model as covariates. Cognitive fatigue rate was used as the primary independent variable in subsequent analyses.

#### Neuropsychological Measures

Neuropsychological data were_inspected for normality and skewed scores were transformed using the Box-Cox method (60). Two scores required this transformation- CMDI Total and STAI State Total. Linear regression analyses were conducted with cognitive fatigue rate as the independent variable and behavioral score as the dependent variable. Sex, education, age, and disease duration were included in the model as covariates. Though MFIS Cognitive and CMDI Total are the primary variables of interest for our paper, additional subscales for these measures have been included for reference.

#### Whole Brain Lesion Volume

Whole brain lesion volumes were normally distributed. Linear regression analyses were conducted with cognitive fatigue rate as the independent variable and lesion volume as the dependent variable. Sex, age, and disease duration were included as covariates.

#### White Matter

To examine the relationship between white matter and cognitive fatigue rate in our MS sample, multiple regression analyses were conducted using FSL's General Linear Model (GLM) Setup utility and TBSS. First, a GLM script was created using the GLM Setup GUI by designating the variable of interest (i.e., cognitive fatigue rate) and covariates of no interest (age, sex, lesion volume, and disease duration). Missing disease duration scores (*n* = 4) were inputted while accounting for age. Two contrasts were included in each design matrix designating 1 or −1 to the variable of interest. This was done to help determine the direction of the relationship between the variable of interest and white matter skeleton. Voxel-wise regression analyses were run on MK, AK, and RK maps using the aforementioned statistical design matrix and FSL's Randomize tool. For the latter, a permutation-based inference (5,000 permutations) correction for multiple comparisons with a Threshold-Free Cluster Enhancement was implemented (Smith and Nichols, 2009). The demean option in Randomize (i.e., -D) was used to demean the data and model in all analyses. Lastly, a family-wise error (FWE) correction was used to correct for multiple comparisons.

The John Hopkins University DTI-based white matter atlases [i.e., ICBM-DTI-81 white matter atlas labels; (61–63)] were used to confirm the location of significant white matter tracts. All results were visualized using FSLeyes.

#### Basal Ganglia Microstructure

Basal ganglia microstructural data was inspected for normality and skewed scores were transformed using the Box-Cox method (60). Linear regression analyses were conducted with cognitive fatigue rate as the independent variable and mean caudate, pallidum, and putamen microstructural value (i.e., mean MK, AK, RK) as the dependent variable. Sex, age, and disease duration were included as covariates.

## Results

### Fatigue Inducing Task Performance and Cognitive Fatigue Rate

Overall, the sample's (*n* = 62) mean total accuracy rate across seven runs of a fatigue inducing task was 87.4% (SD = 15.44, Median = 94.23, Range = 37.14–100). Mean reaction time was 885.3 ms (SD = 235, Median = 844.27, Range = 518.95–1,696.80). After accounting for disease duration, age, and education, results of linear regression analyses showed no significant relationships between cognitive fatigue rate and task accuracy or reaction time.

### Behavioral and Cognitive Measures and Cognitive Fatigue Rate

Descriptive statistics of behavioral and cognitive measures can be found in Table 1 (*n* = 62). Results of a multiple linear regression showed that there was a collective significant effect between sex, age, education, disease duration, and cognitive fatigue rate on MFIS Cognitive score [F_(5,52)_ = 4.02, *p* = 0.004, *R*^2^ = 0.28], with cognitive fatigue rate being the only significant predictor in the model (*t* = 3.20, *p* = 0.002), meaning as trait cognitive fatigue increased, cognitive state fatigue rate also increased. In addition, multiple linear regression results showed that there was a collective significant effect between sex, age, education, disease duration, and cognitive fatigue rate on SDMT performance [F_(5,50)_ = 6.39, *p* < 0.001, *R*^2^ = 0.39], with sex (*t* = 3.92, *p* < 0.001) and years of education (*t* = 2.83, *p* = 0.007) as the significant predictors in the model. Another multiple linear regression showed that there was a collective significant effect between sex, age, education, disease duration, and cognitive fatigue rate on the CMDI Evaluative subscale [F_(5,52)_ = 3.22, *p* = 0.013, *R*^2^ = 0.24], with disease duration (*t* = −2.329, *p* = 0.024) and education (*t* = −3.10, *p* = 0.003) serving as the significant predictors in the model. Models including sex, age, education, disease duration, and cognitive fatigue rate did not significantly predict MFIS Physical, MFIS Psychological, MFIS Total, CMDI Mood, CMDI Vegetative, CMDI Total, STAI State, or STAI Trait. After the *p*-value was adjusted for multiple comparisons, the model predicting MFIS Cognitive and SDMT remained significant.

### Whole Brain Lesion Volume and Cognitive Fatigue Rate

After accounting for age, sex, and disease duration in our sample with usable neuroimaging data (*n* = 56), cognitive fatigue rate was not found to be significantly related to whole brain lesion volume.

### White Matter Microstructure and Cognitive Fatigue Rate

**Figure 1:**
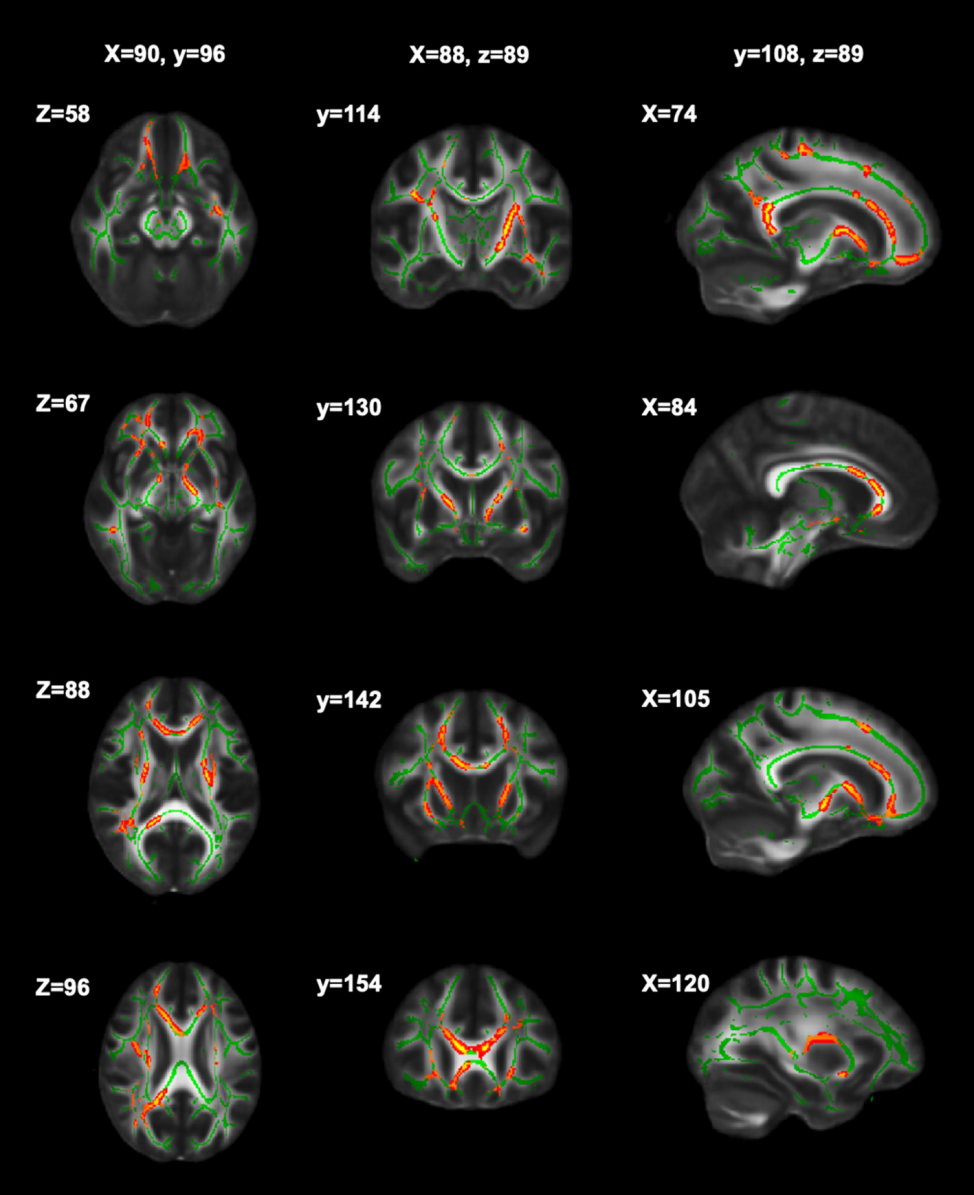
Significant clusters of RK in relation to cognitive fatigue rate. Significant clusters (red) showing where RK is positively related to cognitive fatigue rate (i.e., as RK increases, cognitive fatigue rate increases).

**Figure 2:**
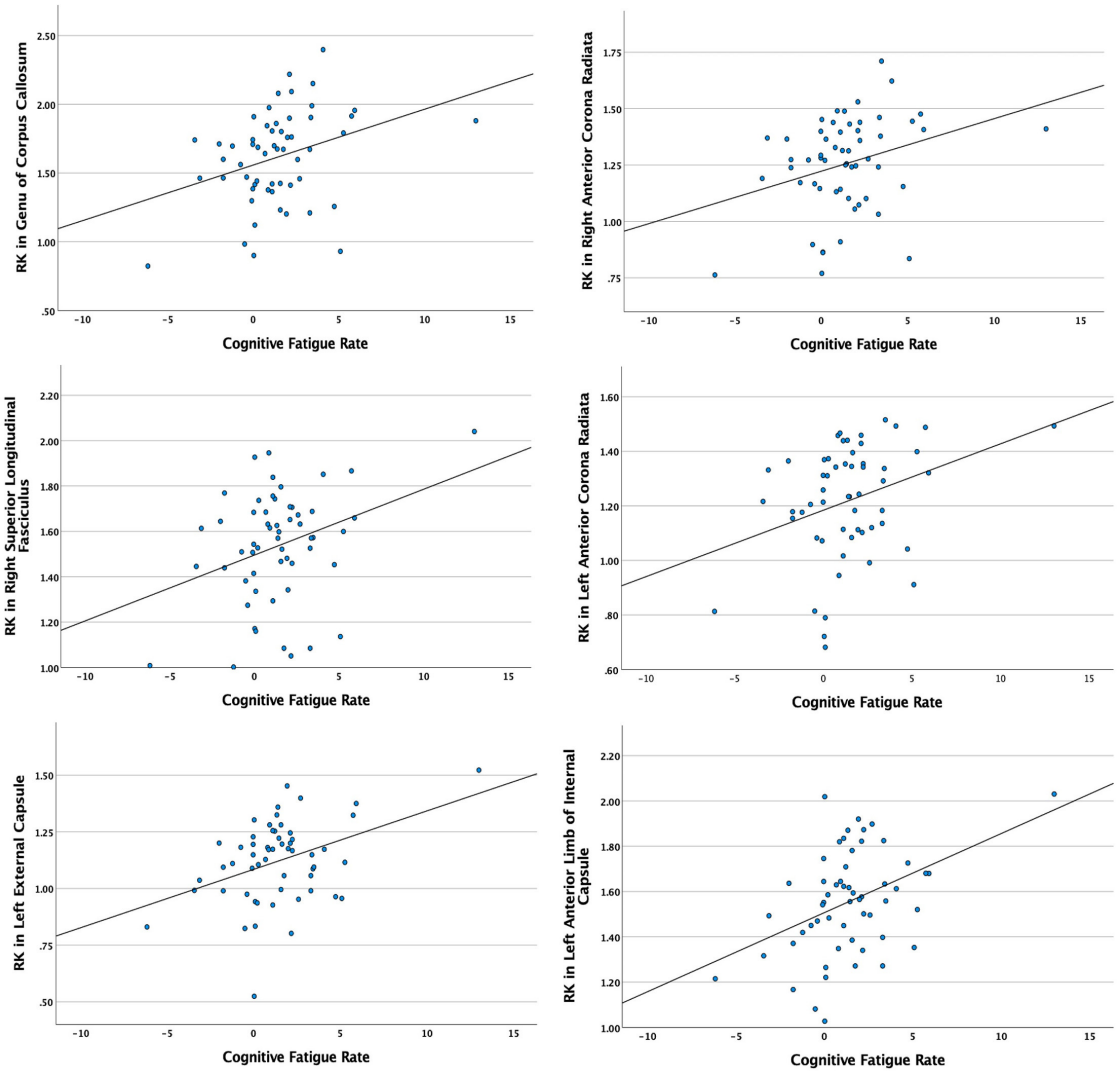
Scatterplots demonstrating the linear relationship between cognitive fatigue rate and radial kurtosis (RK) in tracts with the six greatest volumes of significant clusters. Y-axes = RK in significant white matter tracts. X-axes = cognitive fatigue rate. Analyses were conducted with and without apparent outliers without significant changes in results.

Significant relationships between cognitive fatigue rate and white matter were found only for RK (*n* = 56). RK is a measurement of diffusivity radial to axonal fibers (i.e., perpendicular to the major axis of an axon), with increases in RK suggesting more compromised white matter microstructure. It has been proposed that increases in RK are related to dysmyelination and/or demyelination (65). After accounting for age, sex, lesion volume, and disease duration, results from multiple regression analyses showed cognitive fatigue rate to be positively correlated (*p* < 0.05) with RK in the corpus callosum (genu, body, splenium), anterior corona radiata (left, right), superior longitudinal fasciculus (right), external capsule (left, right), anterior limb of internal capsule (left, right), posterior limb of internal capsule (left, right), superior corona radiata (left, right), posterior thalamic radiation (right), and posterior corona radiata (right). The analyses were run with and without an apparent outlier without differences in results. Thus, presented results include the apparent outlier. Cluster details, including affected white matter tracts and total voxels of significant clusters within these tracts can be found in Table 2. Significant clusters and tracts demonstrating the linear association between RK and cognitive fatigue rate are presented in Figure 1. Plots demonstrating the linear association between RK and cognitive fatigue rate in the six tracts with the greatest volume of significant clusters can be found in Figure 2. Plots for remaining significant tracts (not pictured) show the same graphical pattern as the plots in Figure 2.

**Table 2:** Number of voxels for significant clusters of RK × slope.

**Region name**	**Number of voxels**
Genu of corpus callosum	1,028
Right anterior corona radiata	855
Right superior longitudinal fasciculus	798
Left anterior corona radiata	707
Left external capsule	672
Left anterior limb of internal capsule	573
Splenium of corpus callosum	472
Right anterior limb of internal capsule	471
Body of corpus callosum	436
Left posterior limb of internal capsule	434
Right superior corona radiata	389
Right external capsule	379
Left superior corona radiata	305
Right posterior thalamic radiation	178
Right posterior corona radiata	166
Right posterior limb of internal capsule	125

### Basal Ganglia Microstructure and Cognitive Fatigue Rate

Results of multiple linear regression analyses (*n* = 56) showed a collective significant effect between sex, age, disease duration, and cognitive fatigue rate on putamen RK [F_(4,47)_ = 2.67, *p* = 0.044, *R*^2^ = 0.19]. Individual predictors were examined further and showed that cognitive fatigue rate (*t* = 2.50, *p* = 0.016) was the sole significant predictor in the model. That is, as cognitive fatigue rate increased, RK also increased (i.e., poorer white matter integrity). There was a trend for cognitive fatigue rate being associated with MK in the pallidum after accounting for sex, age, and disease duration [F_(4,47)_ = 2.51, *p* = 0.054, *R*^2^ = 0.18], with cognitive fatigue rate (*t* = 2.08, *p* = 0.043) and disease duration (*t* = −2.20, *p* = 0.033) driving the model. That is, as cognitive fatigue rate increased, MK in the pallidum also increased (i.e., poorer white matter integrity). No models were significant for the caudate (MK, AK, RK) or aspects of the pallidum (AK, RK) and MK or AK of the putamen. After the *p*-value was adjusted for multiple comparisons, the models predicting putamen RK and pallidum MK were no longer significant.

## Discussion

The current study examined the relationship between cognitive fatigue rate and white matter and basal ganglia microstructure using advanced diffusion imaging in a group of pwMS. Our primary aim was to identify potential neural correlates that relate to how quickly or slowly a pwMS becomes cognitively fatigued. In addition, we investigated how cognitive fatigue rate relates to whole brain lesion volume, performance during a fatigue inducing task (i.e., RT, accuracy), and neuropsychological measures. We found cognitive fatigue rate to be related to several white matter tracts (i.e., corpus callosum, anterior corona radiata, superior longitudinal fasciculus, external capsule, anterior and posterior limb of the internal capsule, superior and posterior corona radiata, posterior thalamic radiation), with many having been associated with basal ganglia connectivity or the previously proposed “fatigue network” (28). In addition, cognitive fatigue rate was associated with the microstructure within the putamen and pallidum (trend), though these did not survive multiple comparisons correction. Lastly, cognitive fatigue rate was found to be associated with trait cognitive fatigue, but not depression, anxiety, whole brain lesion volume, SDMT performance, or performance during a fatigue inducing task (i.e., RT, accuracy). The latter is consistent with previous examinations showing that performance measures, such as reaction time and accuracy, are weakly correlated with fatigue ratings (66–68).

One important finding from the current study is lower white matter integrity is associated with a faster onset of state fatigue (i.e., steeper cognitive fatigue rate), such that when white matter integrity is lower, pwMS show a faster onset of cognitive fatigue. Many of the tracts identified in our study have diffuse connections with brain areas that have been associated with fatigue in MS, including the thalamus, basal ganglia (e.g., caudate, putamen, ventral striatum), and frontal cortical areas. It has been suggested that disruption in networks that connect the cortex, particularly the frontal cortex, with deep gray matter areas such as the basal ganglia and thalami is what drives fatigue (13, 14, 69). Thus, our findings of compromised white matter along tracts that are associated with fatigue networks, such as the internal/external capsules and corona radiata, provide initial evidence that these networks may also be involved in the development and experience of fatigue over time (i.e., cognitive fatigue rate).

Many of the white matter tracts found to be significantly associated with cognitive fatigue rate are consistent with other investigations of fatigue in MS. However, it should be noted that no other study to date has looked at cognitive fatigue *rate* in relation to structural brain outcomes. Nonetheless, the congruence of our findings with previous studies broadens our understanding of the structural neural correlates underlying the multifaceted characteristics of fatigue in MS. Tracts identified in our study, including the internal capsule, external capsule, corpus callosum, and corona radiata have all been linked to fatigue in MS (17, 18, 70, 71) and non-MS populations (21, 72). Other studies also identified additional tracts not significant in our analysis, which may be due to differing methodologies or our approach to quantifying fatigue (i.e., cognitive fatigue rate). Given we focused on state cognitive fatigue rate, rather than trait fatigue, the tracts identified in our analysis may be specific to the temporal properties of the onset and progression of fatigue over time, which offers a unique perspective to how we think about and study fatigue in MS.

Given the role of the basal ganglia in previous investigations of fatigue, we thought it important to also examine the microstructure of basal ganglia structures in relation to cognitive fatigue rate. We did find relationships between putamen (RK) and pallidum (MK; trend) and cognitive fatigue rate, but they did not survive multiple comparison correction. This may be due to the methodology used or the size of our sample. Regardless, previous studies examining the structural and functional properties of basal ganglia structures have demonstrated a significant role of the basal ganglia to fatigue in MS (22, 23, 25, 26, 28), and our results suggest that future studies should investigate the relationship between the microstructure of the basal ganglia and cognitive fatigue.

Additionally, we found a positive association between cognitive fatigue rate and trait cognitive fatigue, as measured by the MFIS. To date, studies examining the relationship between state and trait fatigue have been mixed, with some studies demonstrating no relationship, while others show a small to medium relationship (12, 73, 74). Though we did find a significant association, it is notable that the amount of variance shared by the two variables was small (28%), suggesting that while state and trait fatigue both measure aspects of fatigue, the constructs they measure appear to differ considerably. The way in which state and trait fatigue are measured is also important to consider. In the current study, we took a novel approach by not only examining state fatigue during a fatigue inducing task, but we examined the rate at which pwMS became fatigued. Thus, it is likely we are capturing an aspect of fatigue that is missed by trait fatigue measures. Understanding these differentiations will be crucial for delineating cognitive fatigue and how best to measure it.

Our examination of microstructural neural correlates in relation to cognitive fatigue rate fills a gap in the current literature by showing how possible weakening of white matter pathways impacts the development of fatigue over time. Though investigations of trait fatigue measures have laid the foundation for our understanding of fatigue in MS, they fall short in their ability to adequately capture the in-the-moment experience of fatigue. Our study aimed to remedy this shortcoming by using a state fatigue measure that allowed us to calculate fatigue over time. The identification of white matter tracts related to cognitive fatigue rate has clinical implications, since disruptions of white matter tracts may contribute to dysregulation in previously established “fatigue networks.” Thus, by understanding the structural connectivity underlying fatigue-associated brain functioning, we can develop interventions that modulate these fatigue networks.

## Limitations and Future Directions

Though our study produced important and novel results, several limitations exist. First, our sample size was relatively small and limited to individuals with a relapsing-remitting MS disease course, thereby impacting our statistical power and ability to generalize our results to more progressive subtypes. In addition, we did not have access to certain disease-related variables or pertinent comorbidities, such as EDSS, DMT, and sleep disorders/sleepiness and therefore could not determine how these variables may have played a role in our results. Our sample was largely white and highly educated which does not represent the diversity of individuals with MS and limits our ability to generalize our results to the larger MS community. Future studies should make it a priority to recruit more diverse samples to better understand how fatigue impacts individuals from more diverse backgrounds. Next, our analyses of brain metrics were restricted to individual tracts and brain areas, meaning we did not investigate neural network properties. Thus, future work would benefit from not only replicating the results of the current study with a larger, more diverse sample, but also incorporating network-based analyses (e.g., graph theory) to better understand the structural brain networks underlying rate of fatigue. Lastly, while within group studies have many benefits, they also carry limitations. As such, the current study is limited regarding the conclusions that can be made from the results. Future directions include the collection and inclusion of control data to conduct group comparisons.

## Conclusion

Our results show the relationship between cognitive fatigue rate and microstructural properties in the white matter and in the basal ganglia in MS. We identified white matter tracts that connect brain areas that have been associated with fatigue (e.g., frontal cortical areas, thalami, basal ganglia), showing that specific white matter disruptions may be contributing to the rate at which pwMS become fatigued. Our approach of using cognitive fatigue rate, rather than trait fatigue, brings us closer to understanding how brain pathology may be impacting the experience of fatigue in the moment, which is crucial for developing interventions. These results hold promise for continuing to unpack the complex construct that is fatigue.

## Data Availability Statement

The raw data supporting the conclusions of this article will be made available by the authors, without undue reservation.

## Ethics Statement

The studies involving human participants were reviewed and approved by Kessler Foundation Institutional Review Board. The patients/participants provided their written informed consent to participate in this study.

## Author Contributions

CR, GW, JD, and BY contributed to the conceptualization of the manuscript. CR wrote the main manuscript. GW, JD, and BY reviewed and edited the manuscript. All authors contributed to the article and approved the submitted version.

## Funding

We would like to acknowledge grant support from the National Multiple Sclerosis Society (RG-1701-26930 to BY) and Kessler Foundation.

## Conflict of Interest

CR, GW, JD, and BY were employed by Kessler Foundation. The authors declare that the research was conducted in the absence of any additional commercial or financial relationships that could be construed as a potential conflict of interest.

## Publisher's Note

All claims expressed in this article are solely those of the authors and do not necessarily represent those of their affiliated organizations, or those of the publisher, the editors and the reviewers. Any product that may be evaluated in this article, or claim that may be made by its manufacturer, is not guaranteed or endorsed by the publisher.
